# *ETX1* is over-expressed in the glaucomatous trabecular meshwork

**Published:** 2009-10-16

**Authors:** Saradha Iragavarapu, Mabel E. Algeciras, Richard K. Lee, Sanjoy K. Bhattacharya

**Affiliations:** Bascom Palmer Eye Institute, University of Miami, Miller School of Medicine, Miami, FL

## Abstract

**Purpose:**

To determine whether exon-trapped X chromosome clone 1 (*ETX1*) is overexpressed in the trabecular meshwork (TM) of glaucomatous human eyes compared to controls.

**Methods:**

Immunohistochemical, western blot, and enzyme-linked immunosorbent assay analysis were used with human tissues and TM protein extracts. Reverse transcription-PCR was performed on isolated mRNA-derived cDNA preparations.

**Results:**

Elevated expression levels of *ETX1* were detected in glaucomatous compared to control TM tissue. This corroborates previous detection of *ETX1* in glaucomatous TM by proteomic analysis. *ETX1* mRNA is present in TM tissue, suggesting ETX1 protein is locally produced within TM cells.

**Conclusions:**

This is the first report demonstrating overexpression of *ETX1* in glaucomatous TM. *ETX1* expression may regulate TM protein interactions involved in cell adhesion, and its aberrant overexpression may be part of the pathophysiological pathway in the development of glaucoma.

## Introduction

The glaucomas are a group of neurodegenerative ocular diseases that affect over 70 million individuals worldwide [1]. Glaucoma is characterized by irreversible damage to the optic nerve and retinal ganglion cell death. Static perimetry is used to determine visual field loss in the clinical assessment of glaucoma [2,3]. The glaucomas are termed primary when the etiology of optic nerve damage is unknown and secondary when a mechanism for elevated intraocular pressure (IOP) can be ascribed to the development of glaucoma. Primary open angle glaucoma (POAG) is typically associated with elevated IOP, which occurs secondary to an imbalance between aqueous humor production and outflow [4]. Aqueous humor is a clear liquid secreted into the posterior chamber, is actively produced by the ciliary epithelium, and exits through the anterior chamber into Schlemm’s canal. Aqueous humor provides vital nutritional support to anterior chamber tissues, including the lens and the cornea. Aqueous humor outflow experiences most resistance at a filter-like structural region termed the trabecular meshwork (TM). In glaucoma, resistance to outflow at the level of the TM is substantially increased, resulting in increased IOP [5].

Exon-trapped X chromosome clone 1 (*ETX1*), a protein of unknown function, was previously localized in retina and identified as a candidate disease gene responsible for X-linked retinitis pigmentosa (xlRP) [6]. This protein is also known as Sushi repeat-containing protein (SRPX; Swiss-Prot accession number P78539 and Unigene identifier Hs. 15154). However due to the presence of mutations in normal populations as well as those with xlRP, gene mutation studies could not conclusively demonstrate that *ETX1* mutations are linked to retinitis pigmentosa [6]. Proteomic analyses of surgical and cadaveric TM demonstrated the presence of ETX1 in glaucomatous but not control TM tissue [7]. This initial identification of ETX1 in TM tissue using proteomic approaches necessitated validation by other molecular methods and determination as to whether the protein is synthesized locally in the TM or transported by aqueous humor to the TM from elsewhere in the anterior segment of the eye.

To determine ETX1 protein localization in the eye and probe its expression levels, we produced polyclonal antibodies against specific ETX1 peptides. Using immunohistochemical, western blot, and enzyme-linked immunosorbent assay (ELISA) techniques, we demonstrated the presence of ETX1 in the TM and retina. Using reverse transcription (RT)-PCR, we demonstrated local expression of *ETX1* messenger RNA along with genetic analysis of *ETX1* in glaucoma and normal donors.

## Methods

### Tissue procurement

Human tissues were derived from donor eyes from normal control and POAG cadavers, enucleated within 8–12 h of death and obtained from the National Disease Research Interchange (Philadelphia, PA). Glaucomatous eyes with documented optic neuropathy and progressive deterioration in the visual field compatible with glaucoma were procured. Control eyes were from normal donors that lacked optic neuropathy and had no history of eye disease. Both control and glaucomatous donors lacked major central nervous system disorders. All eyes used in this study originated from Caucasian donors between 50–90 years of age.

### Western blot analysis

Protein was extracted from tissues by homogenization in 125 mM Tris-Cl buffer pH 7.0 containing 100 mM NaCl and 0.1% sodium dodecyl sulfate (SDS) and 1 mM dithiothreitol. Insoluble material was removed by centrifugation (8,000× g for 5 min), and soluble protein quantified by the Bradford assay [8]. Western blot analyses were performed with 10 µg of protein extract (unless stated otherwise), electroblotting to polyvinylidene fluoride (PVDF) membrane and probing with chicken polyclonal antibody (Aves Lab Inc., Portland, OR) custom produced against CZ SNF PEG DHK IQY TVY DRA ENK (residues #228–248 in the ETX1 protein sequence named hSRPX#3). Secondary goat antichicken IgY was used for detection. Two other antibodies were also made: hSRPX#1 (against peptide CZ DSP LED DEV GYS HPR YKD T; residues #36–54 in the ETX1 protein sequence) and hSRPX#2 (against peptide CP SVK ERI AEP NKL TVR VSW ET; residues #184–205 in the ETX1 sequence), which were also used for ELISA and western blot analysis. For all analyses, commercially available 4–20% SDS-polyacrylamide gels (Invitrogen Inc., Carlsbad, CA) were used unless stated otherwise.

### Enzyme-linked immunosorbent assay analysis

For quantification of ETX1, ELISA was performed following established protocols [9]. TM protein extracts were centrifuged at 10,000x g for 10 min, then transferred to wells in a 96 well plate (Costar 9018 plate; catalog number 44-2504; eBioscience Inc., San Diego, CA), and incubated for 20 min at room temperature. The supernatant was discarded and the plate was washed with PBS. Plates were blocked with 1% BSA for 1 h, washed with PBS and incubated 1 h with ETX1 (hSRPX#3) primary antibody.  Secondary antibody coupled with alkaline phosphatase was used for primary antibody detection with one hour incubation period. The plate was then washed with PBS, incubated with phosphatase substrate (100 µl /well) in diethanolamine buffer pH 7.5 and quantified at 405 nm on a plate reader (BioTek Synergy HT, BioTek Instruments, Inc., Winooski, VT). A similar analysis was performed using a primary rabbit polyclonal glyceraldehyde-3-phosphate dehydrogenase (GAPDH) loading control antibody (Sigma Chemical Co., St. Louis, MO). ETX1 immunoreactivity was normalized to GAPDH immunoreactivity.

### Immunohistochemical analyses

Cadaver eyes were fixed immediately after enucleation with calcium acetate buffered 4% paraformaldehyde and embedded in paraffin. Globes were sectioned (10 µm) and incubated with 5% BlokHen (Aves labs Inc.) in phosphate buffered saline (PBS). They were then first incubated with 10 ng anti-ETX1 (hSRX#3) antibody overnight at 4 °C and subsequently with 10 ng secondary antibody conjugated with CY-5 (Molecular Probes, Invitrogen Corporation, Carlsbad, CA) for 1 h at room temperature. Sections were sealed with Vectashield (Vector Labs, Burlingame, CA) and analyzed with a Leica TSP-5 confocal microscope (Leica, Exton, PA). Five donor tissues each for control and glaucomatous TM were examined. To ensure identical processing and uniform exposure, control and glaucomatous sections were examined side by side on the same slide. A series of 1-µm xy (en face) images were collected and summed for an image representing a three-dimensional projection of the entire 10-µm section. Confocal microscopic panels were composed using AdobePhotoshop 5.5.

### Reverse transcription-PCR analysis

RNA was isolated from human TM lysates using Trizol (Invitrogen), and cDNA was prepared using established protocols. cDNA samples were PCR-cycled at a modified temperature of 47.5 ºC for annealing using primer pair 5’-ACT CAA TGC CCC AGA GAA TG-3’ and 5'-GAG CCG GTA AAG GAG GTT TC-3'. The PCR products were separated on 2% agarose gels at 100 V for 80 min. The expected size of *ETX1* is approximately 220 bp, determined with a 100-bp molecular weight ladder.

## Results

### ETX1 is localized in trabecular meshwork and retina

**Figure 1:**
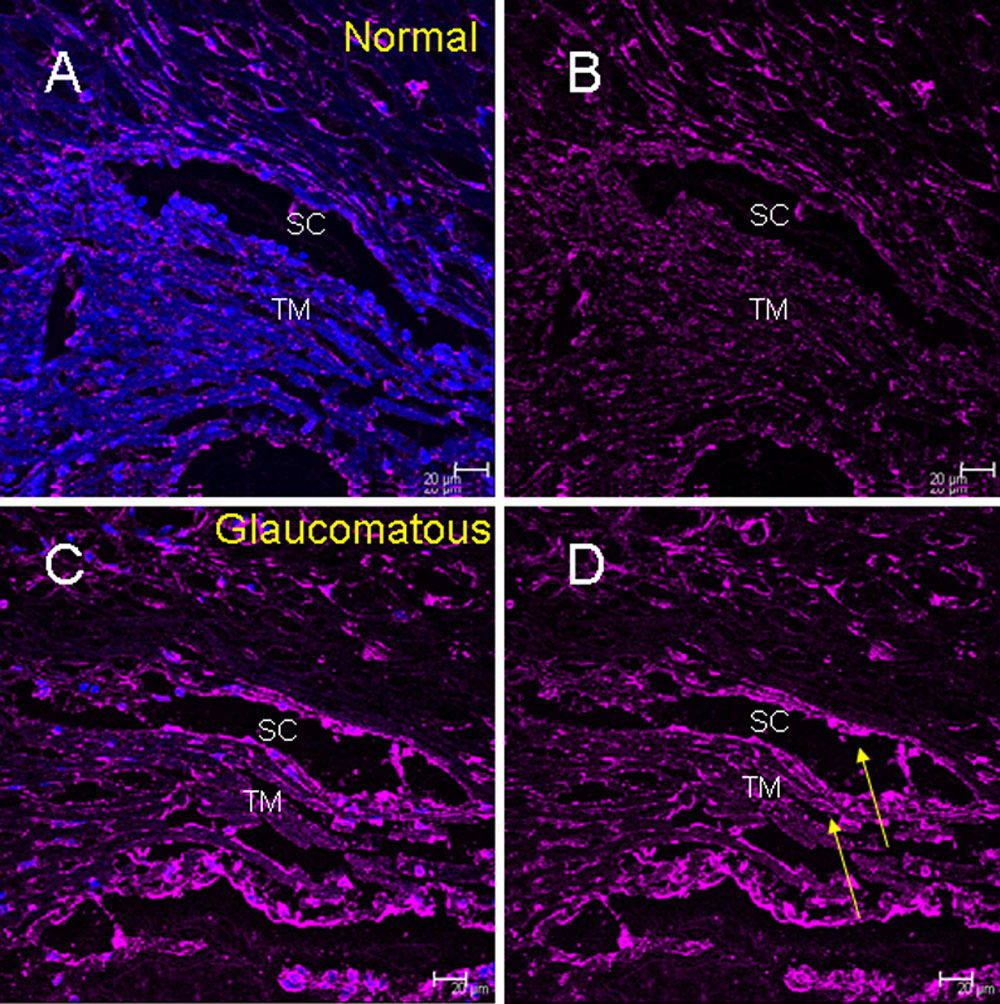
Localization of expression of exon trapped X chromosome clone 1 (ETX1) in human trabecular meshwork. **A**: Merged image of exon trapped X chromosome clone 1 (ETX1)  with secondary Cy5 antibody (magenta) and 4',6-diamidino-2-phenylindole (DAPI) in the normal trabecular meshwork (TM). **B**: The normal TM image stained only for ETX1 (secondary coupled with Cy5; magenta) **C**: The merged image (ETX1 in magenta) in glaucomatous TM as indicated. Glaucomatous TM shows increased expression of ETX1 as compared to normal TM as in **A**. **D**:Glaucomatous TM labeled by ETX1 antibody coupled to Cy5, with arrows indicating areas of hyperfluorescence compared to normal tissue. SC,  Schlemm’s canal.

**Figure 2:**
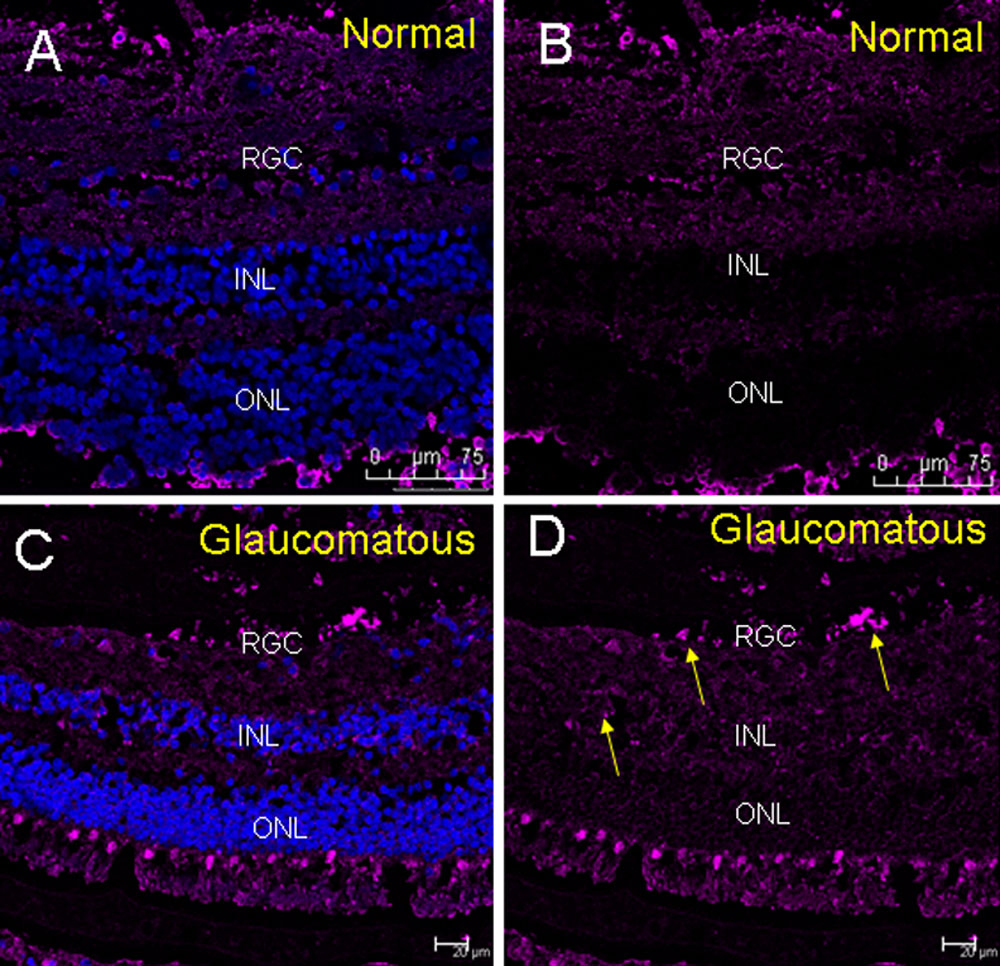
Localization of expression of ETX1 in human retina by immunofluorescence. **A**: Merged image of ETX1 and DAPI in normal retina. Retinal ganglion cell (RGC) layer is intact in the normal retina with good cellularity. **B**: Normal retina stained with anti-ETX1 and secondary antichicken antibody coupled with Cy5. **C**: Merged image of ETX1 and DAPI in glaucomatous retina. RGC layer shows decreased cellularity compared to the normal. **D**: Staining for ETX1 in glaucomatous retina. Clusters of ETX1 indicated with arrows are more frequent and cover more swaths of area in glaucomatous tissue in both the RGC layer as well as diffusely throughout the retina. RGC: Retinal ganglion cell; INL: Inner nuclear layer; ONL: Outer nuclear layer.

Immunohistochemical analysis identified ETX1 protein expression in the TM (Figure 1) and retina (Figure 2). Glaucomatous TM demonstrated elevated ETX1 immunoreactivity compared to normal TM (Figure 1A,B). ETX1 immunoreactivity was visible near Schlemm’s canal and throughout the TM (Figure 1C,D). The normal retina demonstrated more cellular expression within the retinal ganglion cell layer (Figure 2A,B). Glaucomatous retina showed less cellularity as judged by the relative lack of staining with 4',6-diamidino-2-phenylindole (DAPI; Figure 2C), a nuclear marker. However, glaucomatous retina demonstrated large swaths of ETX1 staining at the level of the retinal ganglion cell (RGC Figure 2C,D). These swaths or deposits of ETX1 are more frequent and appeared in larger patches in the glaucomatous retina compared to normal control eyes (Figure 2B,D).

### Elevated expression of ETX1 in glaucomatous trabecular meshwork

**Figure 3:**
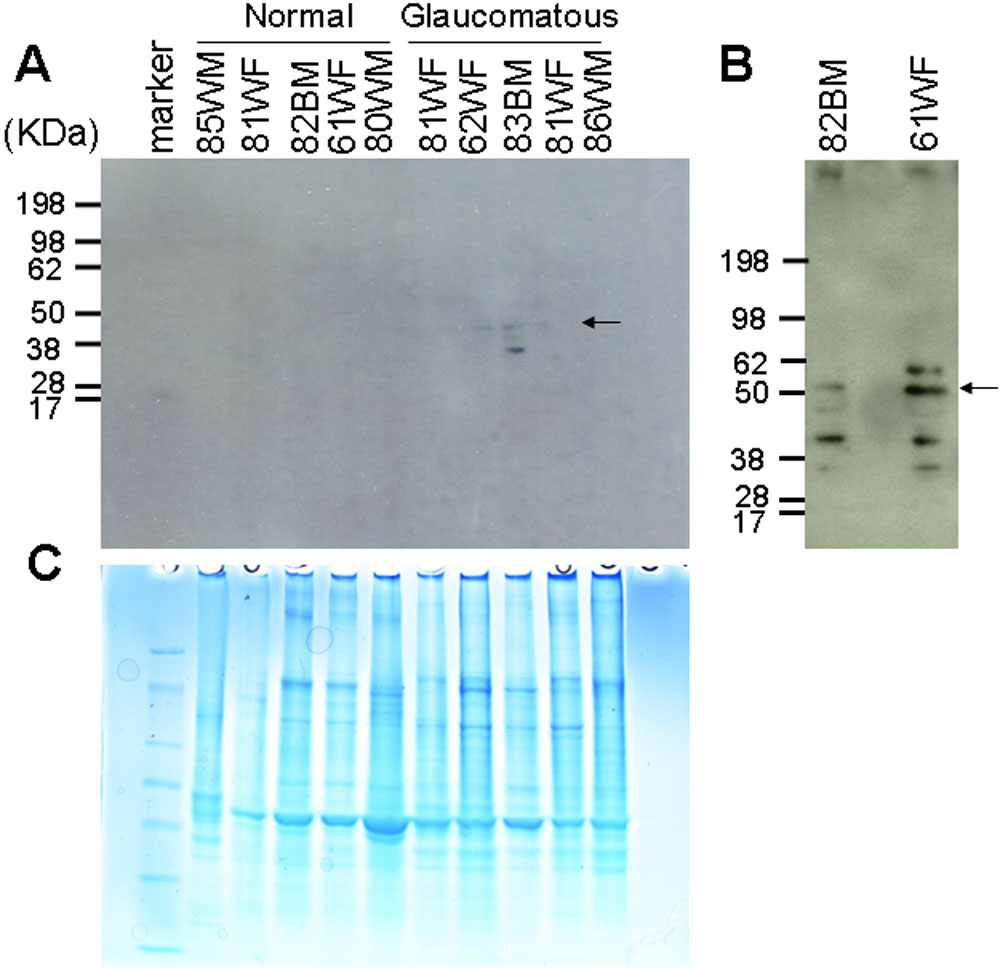
Western blot analysis of trabecular meshwork proteins. TM tissue extract (10 µg) was fractionated on a 4–20% gradient gel (Invitrogen, Carlsbad, CA). **A**: Western blot analysis after transfer onto a polyvinylidene fluoride membrane. The blot was probed with 17–20 µg of chicken polyclonal antibody hSRPX#3 (generated against a synthetic peptide; see methods). **B**: Western blot analysis in normal controls with a protein load of 80 µg and probed with 17–20 µg of chicken polyclonal antibody hSRPX#3. **C**: Identical gel as in **A** stained with Commassie blue R-250.

**Figure 4:**
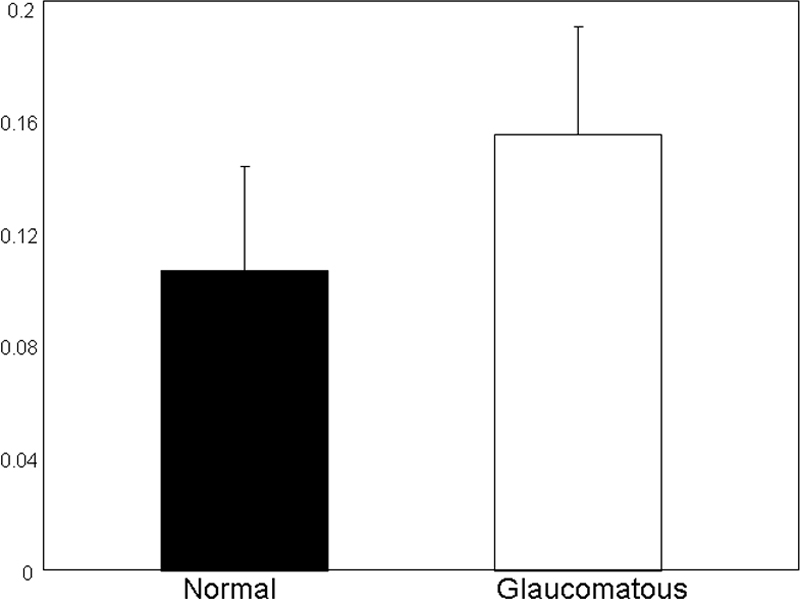
Enzyme-linked immunosorbent assay analysis using anti-ETX1 on proteins from normal and glaucomatous trabecular meshwork tissue. The ETX1 immunoreactivity was normalized to GAPDH immunoreactivity determined in an identical fashion.

ETX protein expression was elevated in the TM of glaucoma donor tissues compared to controls (Figure 3A,C). Using western blot analysis, our antibody detected a protein band of approximately 50 kDa corresponding to ETX1 in the TM. This protein band was more abundant in glaucomatous tissue compared to control tissue (Figure 3A,C). Notably, the ETX1 bands are visible in the normal TM with much higher loading of proteins (Figure 3A,C). ELISA analysis of TM extracts normalized to GAPDH immunoreactivity corroborates this finding (Figure 4). Western blot analysis detected four additional protein bands, and except for one all the other bands were lower in molecular weight and could be proteolytic products or alternatively spliced variants (Figure 3A,C). The protein band slightly greater than 50 kDa could be post-translationally modified or alternatively spliced ETX1 (Figure 3C). However the five immunoreactive western blot bands show variation among individuals and are also expressed among normal controls (Figure 3C).

### ETX1 in the trabecular meshwork is locally formed

**Figure 5:**
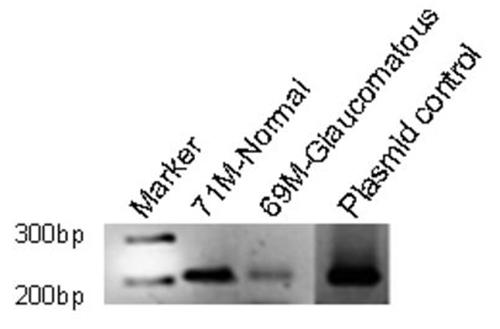
Representative reverse transcription-PCR analysis for *ETX1* in normal and glaucomatous trabecular meshwork tissues. RNA was isolated from human TM lysates, using the Trizol method, and cDNA was prepared using established protocols, as described in the methods section. The PCR products were then run on a 2% agarose gel at 100 V for 80 min. The expected size of *ETX1* is approximately 220 bp.

RT-PCR analysis showed the presence of *ETX1* mRNA in the TM, suggesting local transcription and translation for ETX1. Our primer pair detected a 220 bp fragment, which was found in both normal and glaucomatous TM (Figure 5). This data suggest ETX1 protein expressed in the TM is due to local production and probably not an export product produced by other ocular structures.

## Discussion

*ETX1* mRNA was previously observed to be localized to the retina. *ETX1* mutations were initially identified as being associated with X-linked retinitis pigmentosa (RP), but were not conclusively linked to RP due to their presence in the normal population as well [6,10,11]. Using proteomic analysis we reported the presence of ETX1 protein for the first time in the TM and identified ETX1 protein in the glaucomatous but not normal TM [7]. The reported identification of ETX1 in TM necessitated further validation by independent methods and determination that the protein is locally produced and not accumulated in the TM due to transport from elsewhere. As revealed in our present studies, the previous proteomic detection of ETX1 in the glaucomatous TM is perhaps due to elevated expression of *ETX1* in the TM region and relative low abundance in normal TM (Figure 1C, Figure 3, and Figure 4). ETX1 protein levels are elevated in glaucomatous TM tissue even after correcting expression for the housekeeping protein GAPDH (Figure 4).

ETX1 is a protein of unknown function, contains one hyaline repeat (HYR), and three interrupted complement control protein or sushi repeat domains but lacks C-type lectin or epidermal growth factor domains that exclude the likelihood of the protein being a cell adhesion molecule. A hyaline vascular system has been implicated in eye diseases [12], including glaucoma [13]. In a differential screen, ETX1 has also been found to be differentially expressed in retinal and choroidal microvascular endothelial cells [14]. ETX1 has two spliced isoforms, 78539 and 78539-2. The ETX1 protein is also referred to as Sushi repeat-containing protein. The X-linked (Q4VX66) variant is 464 (451 for isoform 2) amino acids long and has a theoretical molecular weight of 51,572 Da. Theoretical trypsin digestion of either isoform provides about 88.1% coverage, generating identical peptides. Splice variants therefore cannot be readily differentiated using in-gel trypsin digestion and mass spectrometry. Our polyclonal antibody (hSRPX#3), raised in chicken against a synthetic peptide, cannot distinguish between the two isoforms, and therefore multiple western blot protein bands were identified, using our antibody. The protein band corresponding to 50 kDa is the bonafide ETX1 (either isoform); the lower identified molecular weight band may be a proteolytic product or an alternative splice variant (Figure 3C). The single protein band higher than 50 kDa identified in glaucomatous TM tissue can either be a splice variant or a post-translationally modified protein. Other homologues of ETX1, notably SRPX2, have been implicated to have a role in speech and cognition, while mutations in this gene have been associated with brain disorders [15]. ETX1 has been conjectured to be involved in phagocytosis during disk shedding, cell adhesion to cells other than the pigment epithelium, or signal transduction and has been localized to the surface of photoreceptor cells [6,10]. A high degree of sequence conservation exists among Sushi repeat proteins across different mammalian species, with over 89% identity and 92% similarity between mouse, rat, bovine, and human. Differentially spliced isoforms of Sushi repeat-containing proteins have amino acid lengths ranging between 358 and 459 (Table 1). The complement control protein (CCP) module in ETX1 is implicated in protein–protein interactions [16] mediating complement component interactions. Complement component proteins have been shown to play a role in glaucoma [17] and members of complement components have been detected in glaucomatous TM [7,18]. *ETX1* is possibly upregulated in glaucomatous TM and accumulates due to dysregulation of complement components in glaucoma. ETX1 is possibly involved in regulation of the cell adhesion in the TM, but dysregulation of *ETX1* expression occurs in glaucomatous TM, leading to its accumulation and potential aberrant TM cell adhesion that may lead to increased resistance to aqueous outflow. The ETX1-like proteins have been reported to have tumor suppressor activity [19] and have been found to be involved in growth inhibition. If ETX1 is also contributory to growth inhibition, then that property of ETX1, when it is overexpressed, will be potentially relevant in terms of contributing to decreased cellularity in the glaucomatous TM. Future work in this direction will demonstrate the function of ETX1 in TM tissue and whether mutations in the ETX1 protein impair the fine regulation of the complement system in glaucomatous TM, thereby playing a pathophysiological role in the development of glaucoma.

**Table 1:** Homologues of ETX1 protein in mammals.

**Protein name**	**UniProt accession**	**Species name**	**Amino acid identity (%)**	**Amino acid similarity (%)**	**Length (amino acids)**
Sushi repeat-containing protein SRPX precursor	P78539	*Homo sapiens*	100	100	464
Sushi-repeat-containing protein, X-linked	Q4VX66	*Homo sapiens*	100	100	464
Sushi-repeat-containing protein SRPX precursor	Q9R0M3	*Mus musculus*	92	95	464
Sushi repeat-containing protein SRPX precursor	Q63769	*Rattus norvegicus*	93	95	464
Sushi repeat-containing protein SRPX precursor (DRS protein) (Down- regulated by v-SRC).	UPI0000504721	*Rattus norvegicus*	94	97	460
Hypothetical protein	Q32KV1	*Bos taurus*	90	94	459
Sushi repeat-containing protein SRPX precursor (DRS protein) (Down- regulated by v-SRC).	UPI0000504720	*Rattus norvegicus*	94	97	452
Splice isoform 2 of P78539	P78539-2	*Homo sapiens*	100	100	451
Sushi repeat-containing protein SRPX precursor (DRS protein) (Down- regulated by v-SRC).	UPI0000504722	*Rattus norvegicus*	91	93	449
Splice isoform 2 of Q63769	Q63769-2	*Rattus norvegicus*	89	92	444
PREDICTED: similar to Sushi repeat-containing protein SRPX precursor, partial	UPI00005C0986	*Bos taurus*	89	93	358
